# Personality and orthorexia-related eating attitudes in young adults

**DOI:** 10.1590/1806-9282.20260971

**Published:** 2026-11-09

**Authors:** Nur Demirbas, Kezban Elif Çayır, Nazan Karaoglu

**Affiliations:** 1 Necmettin Erbakan University, Faculty of Medicine, Department of Family Medicine – Konya, Türkiye.

**Keywords:** Orthorexia nervosa, Feeding behavior, Personality, Neuroticism, Extraversion, Young adult

## Abstract

**OBJECTIVE:**

The aim of this study is to examine the associations between Eysenck personality dimensions and orthorexia-related eating attitudes among young adults and to describe the distribution of these attitudes across sociodemographic and nutrition-related characteristics.

**METHODS:**

This cross-sectional online survey collected anonymous data from 436 young adults aged 19–25 years between May and July 2022. Orthorexia-related eating attitudes were measured with the Turkish ORTO-11 (lower scores indicate stronger tendencies) and analyzed as a continuous outcome. Personality was assessed with the abbreviated Eysenck Personality Questionnaire–Revised. Multiple linear regression examined independent associations, adjusting for sex and body mass index.

**RESULTS:**

The mean ORTO-11 score was 26.57±5.05. Women scored lower than men (26.25±4.86 vs. 27.41±5.48; p=0.033). In the adjusted model, higher neuroticism was associated with lower ORTO-11 scores (β=-0.11; p=0.024) and higher extraversion with higher scores (β=0.12; p=0.009), indicating stronger and weaker orthorexia-related attitudes, respectively. Psychoticism and body mass index were not associated. The model explained only 4.5% of the variance (R2=0.045; adjusted R2=0.033).

**CONCLUSION:**

Neuroticism and extraversion were statistically but weakly associated with orthorexia-related eating attitudes, whereas body mass index showed no independent association. The very low explained variance and the well-documented psychometric limitations of the ORTO-11, which cannot distinguish a healthy interest in nutrition from pathological orthorexia, mean that these findings are exploratory and should not be interpreted as evidence about orthorexia nervosa.

## INTRODUCTION

Healthy eating is generally regarded as a protective behavior, but in some individuals concern about food purity and dietary control becomes rigid, excessive, and functionally impairing. Orthorexia nervosa has been proposed to describe this pathological preoccupation with healthy eating, characterized by strict dietary rules and distress when these rules are violated. Although orthorexia nervosa is not a recognized diagnostic category in the DSM-5, orthorexia-related eating attitudes have attracted growing research attention, particularly among young adults^1^. A central difficulty is distinguishing an adaptive interest in nutrition, sometimes termed healthy orthorexia, from pathological tendencies, and available screening tools differ in their ability to make this distinction^2^.

Reported estimates vary widely, from approximately 7% in general populations to as high as 58% in high-risk groups, largely reflecting the absence of established diagnostic criteria and the heterogeneity of instruments and cut-off values^3-5^. Proportions derived from screening scales should therefore be interpreted as indicators of orthorexia-related attitudes rather than as clinical prevalence.

Previous studies examining the relationship between personality and orthorexia-related eating have produced inconsistent findings, particularly regarding extraversion and psychoticism^2,6,7^. Evidence from young adult populations in Türkiye also remains limited. Available Turkish studies have examined orthorexia-related eating alongside broader constructs such as body image, physical activity, eating attitudes, or healthy orthorexia^8,9^. However, the independent associations of the Eysenck personality dimensions with orthorexia-related eating attitudes after accounting for sex and body mass index (BMI) have received less attention.

Although the ORTO-11 has recognized psychometric and conceptual limitations^10-13^, it was one of the few instruments with a Turkish validity and reliability study available when the data were collected in 2022^14^ and facilitated comparison with earlier Turkish research. To reduce the risk of overinterpretation, ORTO-11 scores were analyzed primarily as a continuous outcome and were interpreted exclusively as indicators of orthorexia-related eating attitudes rather than as a measure of clinical orthorexia nervosa.

Within these constraints, this study aimed to examine whether Eysenck personality dimensions were independently associated with ORTO-11-assessed eating attitudes among young adults after accounting for sex and BMI and to describe the distribution of these attitudes across sociodemographic and nutrition-related characteristics. We hypothesized that higher neuroticism would be associated with stronger and higher extraversion with weaker orthorexia-related eating attitudes.

## METHODS

Study design and participants: This cross-sectional online survey was reported in accordance with the STROBE statement. Data were collected anonymously through Google Forms between May and July 2022, with the link distributed via social media and messaging platforms using convenience and snowball sampling. Eligible participants were 19–25 years old, literate in Turkish, provided informed consent, and completed the questionnaire. Participants were excluded if they fell outside the age range, did not consent, submitted incomplete questionnaires, or reported implausible anthropometric values. The initial sample-size calculation was based on the single-proportion formula, assuming an expected proportion of 50%, a 95% confidence level, and a 5% margin of error. This calculation yielded a minimum sample of 384 participants; allowing for incomplete or ineligible responses, the final sample comprised 436 participants. Because multiple linear regression constituted the primary multivariable analysis, a sensitivity power analysis was additionally conducted for a fixed model with five predictors. With 436 participants, five predictors, α=0.05, and 80% power, the minimum detectable effect size was Cohen's f^2^=0.030.

Ethics: The study was approved by the Non-Interventional Clinical Research Ethics Committee of Necmettin Erbakan University Faculty of Medicine (no. 2022/3848) and conducted in accordance with the Declaration of Helsinki. All participants provided informed consent, and no identifying information was collected.

Measures: A 20-item researcher-developed form recorded sociodemographic and health-related data, including age, sex, education, employment, marital status, income, social media use, perceived health, physical activity, and dietary behaviors. Regular physical activity was assessed using a single self-report question: "Do you regularly engage in physical activity?" The response options were "yes," "no," and "sometimes." No standardized physical activity instrument was used, and the frequency, duration, and intensity of activity were not assessed. Therefore, the responses reflect participants’ subjective reports of regular physical activity rather than guideline-based physical activity levels. BMI was calculated from self-reported weight and height and categorized using World Health Organization criteria.

Orthorexia-related eating attitudes were assessed with the Turkish version of the ORTO-11^13,14^, an 11-item scale (four-point response format; item 6 reverse-scored) with total scores from 11 to 44, where lower scores indicate stronger orthorexia-related attitudes; it was analyzed primarily as a continuous variable. Cronbach's α was 0.658 in the present sample, indicating modest internal consistency; the corresponding value reported in the Turkish validation study was 0.620^14^. Importantly, the ORTO-15 and its derivatives, including the ORTO-11, have well-documented psychometric and conceptual limitations and do not distinguish a healthy interest in nutrition from pathological orthorexia nervosa^10-12^. Results are therefore framed as orthorexia-related eating attitudes rather than clinical orthorexia. For descriptive purposes only, participants below the sample 25th percentile were labeled as having higher-tendency attitudes; this is a within-sample heuristic, not a diagnostic or prevalence threshold.

Eysenck's Personality Questionnaire-Revised (EPQ-R): Personality dimensions were assessed using the Turkish version of the EPQR-A^15^. The instrument consists of 24 items distributed equally across four six-item subscales: extraversion, neuroticism, psychoticism, and lie/social desirability. Items have a dichotomous "yes/no" response format and are scored as 1 or 0 according to the scoring key. Items 5, 7, 10, 17, and 19 of the lie subscale; items 15 and 20 of the extraversion subscale; and items 3, 16, and 22 of the psychoticism subscale are reverse-coded. Item scores are summed within each subscale, producing possible scores ranging from 0 to 6; higher scores indicate a stronger expression of the corresponding dimension. Higher extraversion scores indicate greater sociability and assertiveness, higher neuroticism scores indicate greater emotional instability and vulnerability to negative affect, and higher psychoticism scores indicate lower conformity and greater interpersonal detachment. The lie subscale is intended to assess socially desirable response tendencies; it was not included as a predictor in the regression model^15^.

Statistical analysis: Continuous variables are presented as mean±standard deviation and categorical variables as frequencies. Normality was assessed visually and with the Kolmogorov-Smirnov test. Independent-samples t-tests, one-way ANOVA, and chi-square tests were used as appropriate. Because some comparison groups were small and markedly unequal in size, Welch's analysis of variance was used for comparisons involving three or more groups. A sensitivity analysis based on the noncentral F distribution was used to determine the minimum effect size detectable by the regression model with 80% power. The primary outcome, the continuous ORTO-11 score, was modeled with multiple linear regression including neuroticism, extraversion, psychoticism, BMI, and sex, selected on theoretical grounds. Model fit was assessed with R^2^ and adjusted R^2^, and multicollinearity with variance inflation factor and tolerance. Because lower ORTO-11 scores indicate stronger orthorexia-related attitudes, negative coefficients denote stronger tendencies. A two-sided p<0.05 was considered significant.

## RESULTS

The 436 participants had a mean age of 20.89±1.93 years, and 72.9% (n=318) were women. Mean BMI was lower in women than in men (21.17±3.10 vs. 23.89±3.80 kg/m^2^; p<0.001). General health was rated as good by 58.3%, 25.9% perceived their diet as healthy, and 26.6% reported regularly checking food labels. The mean ORTO-11 score was 26.57±5.05 (range 15–41). The 25th percentile corresponded to a score of 24, below which 32.6% (n=142) of participants fell. Men scored higher than women (27.41±5.48 vs. 26.25±4.86; p=0.033), indicating relatively weaker orthorexia-related attitudes. BMI categories were not associated with ORTO-11 scores (p=0.997). Lower scores were observed among participants who occasionally followed a special diet (23.04±4.41 vs. 27.08±4.96; p<0.001) and who regularly checked food labels (24.73±5.11 vs. 27.78±5.11; p<0.001) (Tables 1 and 2). The mean EPQR-A subscale scores were 3.78±1.74 for extraversion (median=4, range=0–6), 3.82±1.77 for neuroticism (median=4, range=0–6), 1.69±1.17 for psychoticism (median=2, range=0–5), and 3.89±1.55 for lie/social desirability (median=4, range=0–6).

**Table 1:** Sociodemographic characteristics and ORTO-11 scores.

Characteristic		n (%)	ORTO-11, mean±SD	p
Sex				
	Male	118 (27.1)	27.41±5.48	0.033
	Female	318 (72.9)	26.25±4.86	
Education				
	High school	28 (6.4)	26.96±5.06	0.671
	University	408 (93.6)	26.54±5.06	
BMI category				
	Underweight	69 (15.8)	26.62±4.95	0.997*
	Normal weight	286 (65.6)	26.54±5.11	
	Overweight	68 (15.6)	26.57±5.08	
	Obesity	13 (3.0)	26.84±4.66	
Regular physical activity				
	Yes	79 (18.1)	25.65±5.19	0.073
	No	188 (43.1)	27.00±4.84	
	Sometimes	169 (38.8)	26.96±4.18	
Perceived general health				
	Very good/good	312 (71.5)	26.51±4.97	0.704*
	Moderate	111 (25.5)	26.83±5.26	
	Bad/very bad	13 (3.0)	25.61±5.43	

Independent-samples t-test or one-way ANOVA, as appropriate. BMI is categorized per WHO criteria. Lower ORTO-11 scores indicate stronger orthorexia-related eating attitudes. SD: standard deviation; BMI: body mass index.

* Welch's ANOVA was used for comparisons involving three or more groups with markedly unequal group sizes.

**Table 2:** Dietary characteristics and ORTO-11 scores.

		Dietary characteristic	n (%)	ORTO-11, mean±SD	p
Perceives own diet as healthy				
	Yes	113 (25.9)	26.24±5.27	0.583
	No	164 (37.6)	26.87±4.94	
	Not sure	159 (36.5)	26.51±5.02	
Follows a special diet				
	Yes	18 (4.1)	24.27±4.72	<0.001*
	No	375 (86.0)	27.08±4.96	
	Occasionally	43 (9.9)	23.04±4.41	
Checks food labels/ingredients				
	Yes	116 (26.6)	24.73±5.11	<0.001
	No	146 (33.5)	27.78±5.11	
	Sometimes	174 (39.9)	26.78±4.63	

One-way ANOVA. Lower ORTO-11 scores indicate stronger orthorexia-related eating attitudes. SD: standard deviation.

* Welch's ANOVA was used for comparisons involving three or more groups with markedly unequal group sizes.

The multiple linear regression model was statistically significant, F(5,430) =4.007, p=0.001, but explained only 4.5% of the variance in ORTO-11 scores (R^2^=0.045; adjusted R^2^=0.033). Higher extraversion was independently associated with higher ORTO-11 scores (B=0.361, SE=0.138, β=0.124, p=0.009), indicating weaker orthorexia-related eating attitudes. Higher neuroticism was associated with lower ORTO-11 scores (B=-0.310, SE=0.137, β=-0.109, p=0.024), indicating stronger orthorexia-related eating attitudes. Female sex was also associated with lower ORTO-11 scores (B=-1.306, SE=0.577, β=-0.115, p=0.024). Psychoticism and BMI were not independently associated with ORTO-11 scores. No evidence of problematic multicollinearity was observed, with VIF values ranging from 1.012 to 1.157 (Table 3).

**Table 3:** Multiple linear regression for ORTO-11 scores (n=436).

Predictor	B (SE)	β	95%CI	p
Neuroticism	-0.310 (0.137)	-0.109	-0.579 to -0.042	0.024
Extraversion	0.361 (0.138)	0.124	0.089 to 0.632	0.009
Psychoticism	-0.085 (0.206)	-0.020	-0.489 to 0.320	0.681
Body mass index	-0.097 (0.071)	-0.068	-0.237 to 0.043	0.175
Sex (female)	-1.306 (0.577)	-0.115	-2.439 to -0.172	0.024

B: unstandardized coefficient; SE: standard error; β: standardized coefficient; CI: 95% confidence interval for B. Model R2=0.045. Because lower ORTO-11 scores indicate stronger orthorexia-related attitudes, negative coefficients denote stronger tendencies.

## DISCUSSION

Higher neuroticism was associated with stronger, and higher extraversion with weaker, orthorexia-related eating attitudes, whereas BMI showed no independent association. Crucially, the model explained only 4% of the variance, so personality dimensions account for a minimal share of the variability in ORTO-11 scores and are best regarded as marginal correlates rather than meaningful determinants of orthorexia-related attitudes.

Women scored lower than men, suggesting stronger orthorexia-related attitudes, consistent with some^16^ but not all reports; the greater cultural emphasis on diet quality and "clean eating," reinforced by social media exposure, may contribute to this pattern^17^. The neuroticism finding aligns with the view that emotional vulnerability and intolerance of uncertainty may promote dietary control as a coping strategy^2,6,18^, while the social flexibility and positive affect associated with extraversion may reduce rigid adherence to restrictive food rules^19,20^; this association was nonetheless small and should not be interpreted as protective. Psychoticism was unrelated to ORTO-11 scores, consistent with previous inconsistent findings^2^, and BMI was not independently associated, supporting the view that orthorexia-related attitudes relate more to cognitive and emotional patterns around food than to body size^21^, although BMI was derived from self-report.

These results must be interpreted in light of the well-documented limitations of the ORTO-11. The ORTO-15 and its derivatives have been criticized for their weak and unstable factor structures and their inability to distinguish a healthy interest in nutrition from pathological orthorexia^10-12^. The modest internal consistency observed in the present sample (Cronbach's α=0.658) reinforces these concerns. This level of reliability indicates that the ORTO-11 items may not consistently measure a single underlying construct and may introduce measurement error. Such error could have attenuated the observed associations between personality dimensions and ORTO-11 scores, potentially contributing to the small standardized coefficients and the low explained variance of the regression model. It may also limit the reproducibility of the findings across different samples. We therefore frame our results strictly as exploratory associations with ORTO-11-assessed eating attitudes rather than as evidence concerning orthorexia nervosa. The descriptive 25th-percentile classification should likewise be regarded only as a within-sample heuristic.

Conceptually, orthorexia is increasingly viewed as overlapping with obsessive-compulsive, eating-disorder, and perfectionistic dimensions rather than as a clearly distinct entity^20,21^. Our model did not include obsessive-compulsive traits, perfectionism, anxiety, or social media exposure, all strongly implicated in the literature; their omission likely contributes to the low explained variance and constrains interpretation.

From a primary-care perspective, these results argue for caution rather than routine screening. Because personality dimensions explained little of the variance and the ORTO-11 cannot separate adaptive from pathological eating, isolated low scores should not be read as indicating a disorder. Nonetheless, when clinicians encounter young adults whose rigid and distressing preoccupation with food quality impairs daily functioning, it may be useful to consider co-occurring emotional vulnerability and to assess overlapping obsessive-compulsive and eating-disorder features rather than dietary content alone.

Strengths and limitations: This study has strengths, including its focus on young adults, the simultaneous evaluation of multiple Eysenck personality dimensions, and a sample size adequate for the planned analyses. Several limitations should be noted. The cross-sectional design precludes causal inference. Online convenience and snowball sampling probably over-represented health-conscious young adults and university students, limiting generalizability and introducing selection bias. All data, including the height and weight used for BMI, were self-reported. Beyond the instrument and omitted-variable issues noted above, the small explained variance indicates that other psychological, behavioral, and sociocultural factors should be prioritized in future work, ideally using validated instruments that distinguish healthy from pathological orthorexia together with longitudinal designs.

## CONCLUSION

In this sample of young adults, neuroticism and extraversion were statistically but weakly associated with orthorexia-related eating attitudes, while BMI showed no independent association. Given the very low explained variance and the substantial psychometric limitations of the ORTO-11, these findings are exploratory and should not be interpreted as evidence regarding orthorexia nervosa. Future studies using validated, conceptually adequate measures and longitudinal designs are warranted.

## Data Availability

The datasets generated and/or analyzed during the current study are available from the corresponding author upon reasonable request.
